# Direct Identification of Intact Proteins Using a Low-Resolution Mass Spectrometer with CID^n^/ETnoD

**DOI:** 10.1021/jasms.4c00108

**Published:** 2024-06-21

**Authors:** Cheng-Yu Kuo, Yi-Feng Zheng, Wei-Chen Wang, Jie-Teng Toh, Yu-Ming Hsu, Han-Ju Chien, Chih-Jui Chang, Chien-Chen Lai

**Affiliations:** † Institute of Molecular Biology, National Chung Hsing University, Taichung 402, Taiwan; ‡ Department of Biochemical Science and Technology, National Chiayi University, Chiayi 600, Taiwan; § Department of Molecular Biology and Human Genetics, Tzu Chi University, Hualien City 970, Taiwan; ∥ Advanced Plant and Food Crop Biotechnology Center, National Chung Hsing University, Taichung 402, Taiwan; ⊥ Graduate Institute of Chinese Medical Science, China Medical University, Taichung 406, Taiwan; # Doctoral Program in Translational Medicine, National Chung Hsing University, Taichung 402, Taiwan; ∇ Rong Hsing Translational Medicine Research Center, National Chung Hsing University, Taichung 402, Taiwan

**Keywords:** Intact protein, CID, ETnoD, Low-resolution mass spectrometry, Charged-reduced precursor ions

## Abstract

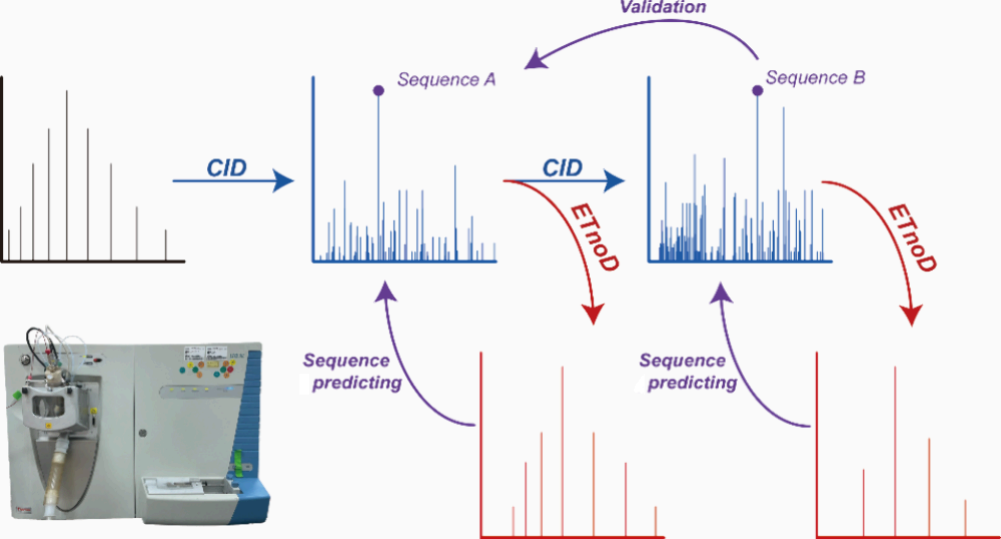

Over the past decades, proteomics has become increasingly important and a heavily discussed topic. The identification of intact proteins remains a major focus in this field. While most intact proteins are analyzed using high-resolution mass spectrometry, identifying them through low-resolution mass spectrometry continues to pose challenges. In our study, we investigated the capability of identifying various intact proteins using collision-induced dissociation (CID) and electron transfer without dissociation (ETnoD). Using myoglobin as our test protein, stable product ions were generated with CID, and the identities of the product ions were identified with ETnoD. ETnoD uses a short activation time (AcT, 5 ms) to create sequential charge-reduced precursor ion (CRI). The charges of the fragments and their sequences were determined with corresponding CRI. The product ions can be selected for subsequent CID (termed CID^n^) combined with ETnoD for further sequence identification and validation. We refer to this method as CID^n^/ETnoD. The use of a multistage CID activation (CID^n^) and ETnoD protocol has been applied to several intact proteins to obtain multiple sequence identifications.

## Introduction

Proteomics has become highly developed in recent years, surpassing genomics due to its attractive and widely useful applications. Along with the concept of the proteome, advances in proteomics have been focused on improving protein identification, leading to increasingly mature and beneficial techniques.^1,2^ The need for protein identification has grown, with applications in both the biopharmaceutical and biomedical fields.^3,4^ Because proteins determine the phenotypes of living beings, the ability to identify proteins is crucial for biomedical advances.^5,6^ An increasing number of disease treatments require the analysis of proteins in patient samples.^7^ Understanding the associations between proteins found in biological samples and disease states can allow improved patient care. The identification of potential disease protein biomarkers is an intense area of research.^8^ Various peptides and proteins have been approved for clinical use by the US Food and Drug Administration,^9^ underscoring the significance of protein identification. Therefore, establishing qualitative methods for quality control is crucial in the area of protein therapeutic research.^10^

In the past decades, the use of mass spectrometry has markedly increased due to its good and reliable evaluation results. Mass spectrometry can be applied to protein identification using either a top-down approach, in which proteins are analyzed intact, or a bottom-up approach, in which proteins are digested prior to analysis.^11^ The approaches typically require the use of high-resolution mass spectrometry, and the resulting spectra are analyzed using commercial software.^12,13^ In general, higher resolution mass spectrometry yields more accurate results, thereby improving protein identification. Protein identification is most commonly performed using ultrahigh-performance liquid chromatography coupled with high-resolution mass spectrometry. Techniques employed include collision-induced dissociation (CID), electron transfer dissociation (ETD), higher-energy collisional dissociation (HCD), and ultraviolet photodissociation (UVPD).^14−19^

CID is among the most common MS/MS techniques used for inducing the fragmentation of peptides or proteins.^20^ CID is used to perform mass spectrometry based quantitative analysis, in which selected reaction monitoring (SRM)^21−23^ is used to evaluate the product ions produced when CID is applied to select precursor ions in specific peptides during tandem mass spectrometry (MS/MS). ETD is a newly introduced mass spectrometry technique that complements CID. During ETD, fluoranthene, a negative ion supplier, reacts with multiple protonated peptides, fragmenting the peptide backbone and causing the cleavage of the N–Cα peptide bond, generating complementary c-type and z-type fragment ions.^24−26^

Understanding the nature of intact proteins is essential for deepening our knowledge of disease states. Achieving full protein sequence coverage and assessing post-translational modifications (PTMs), such as phosphorylation, acetylation, and ubiquitination, are highly sought after in proteomics research.^14−16,27−29^ Prior studies have compared the characteristics of various fragmentation techniques, focusing on aspects such as sequence coverage and PTM (post-translational modification) evaluation. These studies utilized different types of software to identify the optimal methods for analyzing specific proteins. However, while some techniques have been identified as providing high percentages of sequence coverage or detailed insights into PTM locations, most studies have predominantly focused on methods using high-resolution mass spectrometry.

Although several studies have indicated that low-resolution mass spectrometry can be utilized for the quantitation of intact proteins, the validation and identification of these proteins are predominantly performed using high-resolution mass spectrometry.^30^ Although low-resolution mass spectrometry is capable of protein identification by adjusting software parameters, this has only been explored using the bottom-up approach, which limits the identification of intact proteins.

**Scheme 1:**
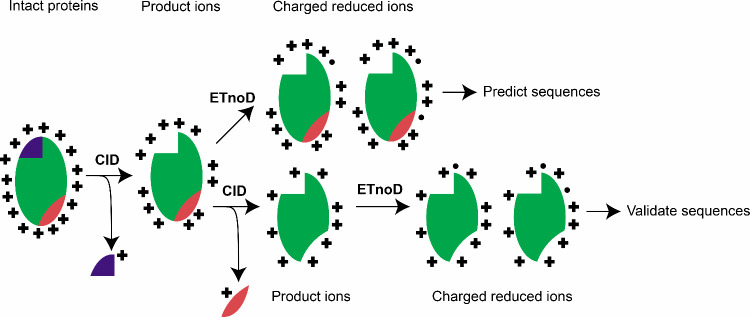
Overview of Multistage Collision-Induced Dissociation/Electron Transfer without Dissociation (CID^n^/ETnoD)

A previous study demonstrated that selected electron transfer reaction monitoring (SETRM) could be applied for the identification and quantitation of doubly charged peptides.^9^ To our knowledge, this study represents the first reported attempt to use CID and ETD for identifying fragments generated from intact proteins. In this study, we employed low-resolution mass spectrometry, specifically using a Thermo LTQ-XL Linear Ion Trap (LTQ-XL), to identify intact proteins. This was achieved by applying a technique combining multistage CID and ETnoD (termed CID^n^/ETnoD, Scheme 1). The generation of stable and reproducible ions from intact proteins was optimized by adjusting electrospray conditions, collision energy (CE), and activation time (AcT). We evaluated the CID^n^/ETnoD method for its capability to predict the charges of intact proteins and to estimate the fragments generated from them. This approach offers a simpler and more direct method for identifying intact proteins compared to current protein identification methods.

## Experimental Section

### Materials and Reagents

Protein standards, including myoglobin (Myo; 17 kDa) from equine skeletal muscle, human recombinant insulin (Insl; 5.8 kDa) with disulfide bonds, lysozyme (Lyso; 14 kDa) from chicken, alpha-lactalbumin (A-lac; 14 kDa) from bovine milk, human hemoglobin (Hemo; 64 kDa), in addition to formic acid (FA) and dithiothreitol (DTT) were purchased from Sigma-Aldrich (St. Louis, MO, USA). The investigated protein standards ranged in molecular weight from 5.8 to 64 kDa. Acetonitrile (ACN), ammonium bicarbonate (ABC), and methanol (MeOH) were purchased from Avantor. Iodoacetamide (IAM) was purchased from GE Healthcare.

### Intact Protein SETRM Method Development and Optimization

#### Sample Preparation and Instrument Parameters

A ThermoFisher Scientific LTQ-XL linear ion trap (San Jose, CA) equipped with a chemical ionization source for the generation of radical ions (fluoranthene) for ETD reactions was used to characterize intact proteins. All intact protein standards were prepared at 1 mg/mL in water and stored at −80 °C until use. Working concentrations of intact proteins standards were prepared through dilution with a mixture of 30% water and 70% ACN containing 0.1% FA (by volume). For both CID and ETD analyses, the precursor ion width was set to 3 Da, and the automatic gain control (AGC) of the precursor cations for the MS^n^ scan was set to 1 × 10^5^. For the CID experiment, the q-value was equal to 0.25, and the normalized CE ranged from 0% to 35%. For the ETD experiment, the reaction time ranged from 3 to 200 ms, and the AGC target for fluoranthene anions ranged from 1 × 10^5^ to 3 × 10^5^.

#### Optimization of CE in CID and AcT in ETD

A variety of CE and AcT values can be used to stabilize the product ion signals from the precursor ions of the intact protein. The linear ion trap (LTQ)-XL with ETD system uses helium as the collision gas and fluoranthene as the electron carrier. SETRM is performed manually by optimizing CE and AcT. The signal for each product ion (MS^2^ and MS^3^) is verified at each step. The optimal CE ranges from 10 to 35, and the optimal AcT ranges from 3 to 100.

#### Stabilization of SETRM in Each Intact Protein

The reproducibility and sensitivity of signals from intact proteins were evaluated rigorously. Each fragmentation procedure used for intact proteins was optimized relative to the signal of the previous spectrum to avoid losing too much signal. The relative standard deviation of each signal obtained from intact proteins was maintained below 15%.

#### Eliminating the Disulfide Bonds of Intact Proteins in Solution

a-lac and Lyso require the elimination of disulfide bonds. DTT and IAM were prepared in 50 mM ABC/ddH_2_O. A-lac and Lyso were diluted to final concentrations of 0.1 mg/mL, and 50 μL of 10 mM DTT was added to each intact protein standard and incubated at 56 °C for 1 h, followed by the addition of 50 μL of 10 mM IAM and incubation at 37 °C for 30 min in the dark.

#### Eradicating the Covalent Bonds of Intact Proteins Using FA Solution

Intact Hemo is too large for analysis and requires the breaking of covalent bonds for detection using our instrument. Intact Hemo was prepared at a concentration of 1 mg/mL in 0.2% FA/ddH_2_O for 1 min,^31^ after which the protein standard was transferred to a new Eppendorf tube and further diluted with a mixture of 30% water and 70% ACN containing 0.1% FA (by volume), as described above.

#### Data Evaluation

The protein sequences of intact proteins were obtained from UniProt. LTQ-XL, and the data are analyzed manually during infusion. The multiple-charged fragments produced from the intact proteins are identified manually. The molecular weights of each intact protein following fragmentation were analyzed using PeakView Software (version 2.1.0.11041).

#### LC-MS/MS Analysis

Five intact protein standards were combined into a mixture with the following composition: 20 ppm Lyso, 50 ppm A-Lac, 50 ppm Insl, 100 ppm Myo, and 100 ppm of Hemo. An Agilent 1200 series HPLC coupled with an LTQ ^XL^ mass spectrometer was used to analyze the mixture. The mobile phases A and B were 0.1% formic acid in H_2_O and 0.1% formic acid in acetonitrile, respectively. The separation was achieved using a Symmetry C4 Column (300 Å, 5 μm, 2.1 mm × 150 mm) with a 28 min gradient. The flow rate was set at 0.4 mL/min. The gradient steps were as follows: 0–1.35 min, 25% B; 1.35–21.25 min, 25–85% B; 21.25–22.25 min, 85% B; 22.25–22.3 min, 85–25% B; 22.3–28 min, 25% B. The injection volume was 5 μL.

## Results and Discussion

### CID and ETD Applied to Intact Myo

**Figure 1:**
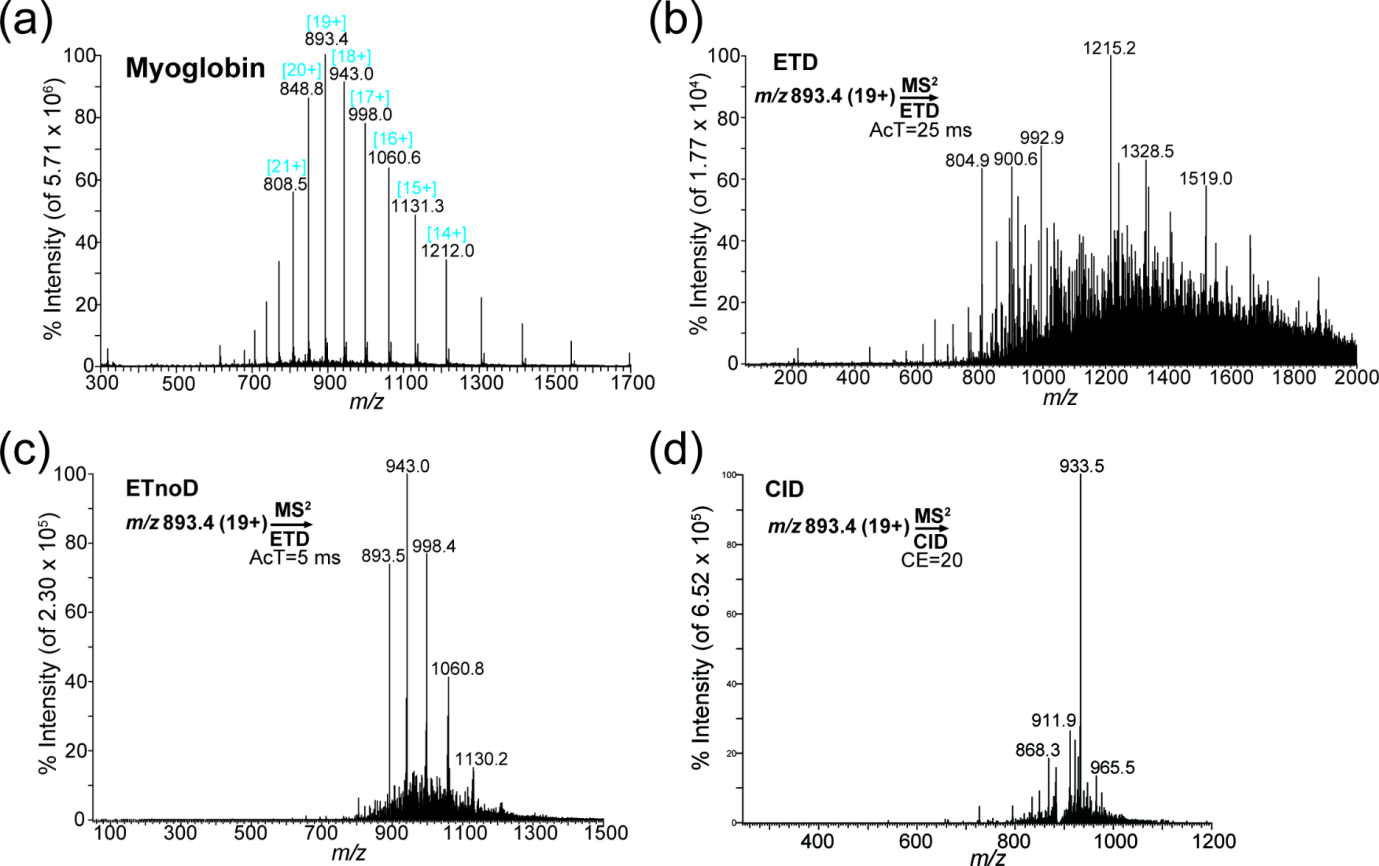
(a) Intact myoglobin charge state distribution generated by electrospray ionization and collected by full-scan mode. The highest intensity charge state [19+] was chosen as the precursor ion. (b) Product ion scan at activation time (AcT) = 25 ms (abundant ions are observed). (c) Product ion scan at AcT = 5 ms (CRI observed). (d) Product ion scan at collision energy (CE) = 20 (one major stable peak observed). CID, collision-induced dissociation; ETD, electron transfer dissociation; ETnoD, electron transfer without dissociation; MS, mass spectrometry.

Before identifying intact proteins, it is necessary to investigate their full-scan spectrum. Identifying reproducible and unique product ions that represent the protein of interest is essential for generating a SETRM transition. Figure 1a shows the intact Myo charge state distribution generated from the electrospray ionization (ESI) source and measured in full-scan mode using LTQ-MS. The highest intensity ion (*m*/*z* 893.4 [19+]) was selected to serve as the precursor ion for further fragmentation, method development, and optimization.

First, ETD was applied to the precursor Myo ion (*m*/*z* 893.4 [19+]) at an AcT of 25 ms (Figure 1b). The product ions generated from ETD were broadly distributed, with no major peaks. However, a shorter AcT (AcT = 5 ms) during fragmentation produces a similar spectral pattern as the full-scan spectra for intact Myo (Figure 1c). A shorter AcT prevents the precursor Myo protein from being completely fragmented, generating CRIs, which is referred to as ETnoD. Comparing the results in Figure 1a and c shows that many peaks appeared similar, indicating that Figure 1c generated CRI from the intact protein. The charge number for intact Myo can be determined by analyzing the two nearby peaks, using the formula 943.0x–x = 998.4 (x–1)- (x–1), where x = 18 (*m*/*z* 943.0 [18+]), representing the charge number of Myo ions (*m*/*z* 943.0).

During CID, the product ions were first generated from the precursor Myo ion (*m*/*z* 893.4 [19+]) at a CE of 20 (Figure 1d). A major product ion (*m*/*z* 933.5) was identified. However, the product ions generated by ETD differed significantly. These results indicate that the product ions generated by ETD (Figure 1b) exhibited more fragment signals compared to those generated by CID (Figure 1d), causing the total ion intensity to be distributed among these signals. Consequently, the ETD method results in a spectrum that is more difficult to interpret. On the other hand, CID generats a single major peak. Therefore, we selected CID as the fragmentation method due to its ability to produce stable, major fragmented ions.

### CID/ETnoD Applied to Intact Myo

**Figure 2:**
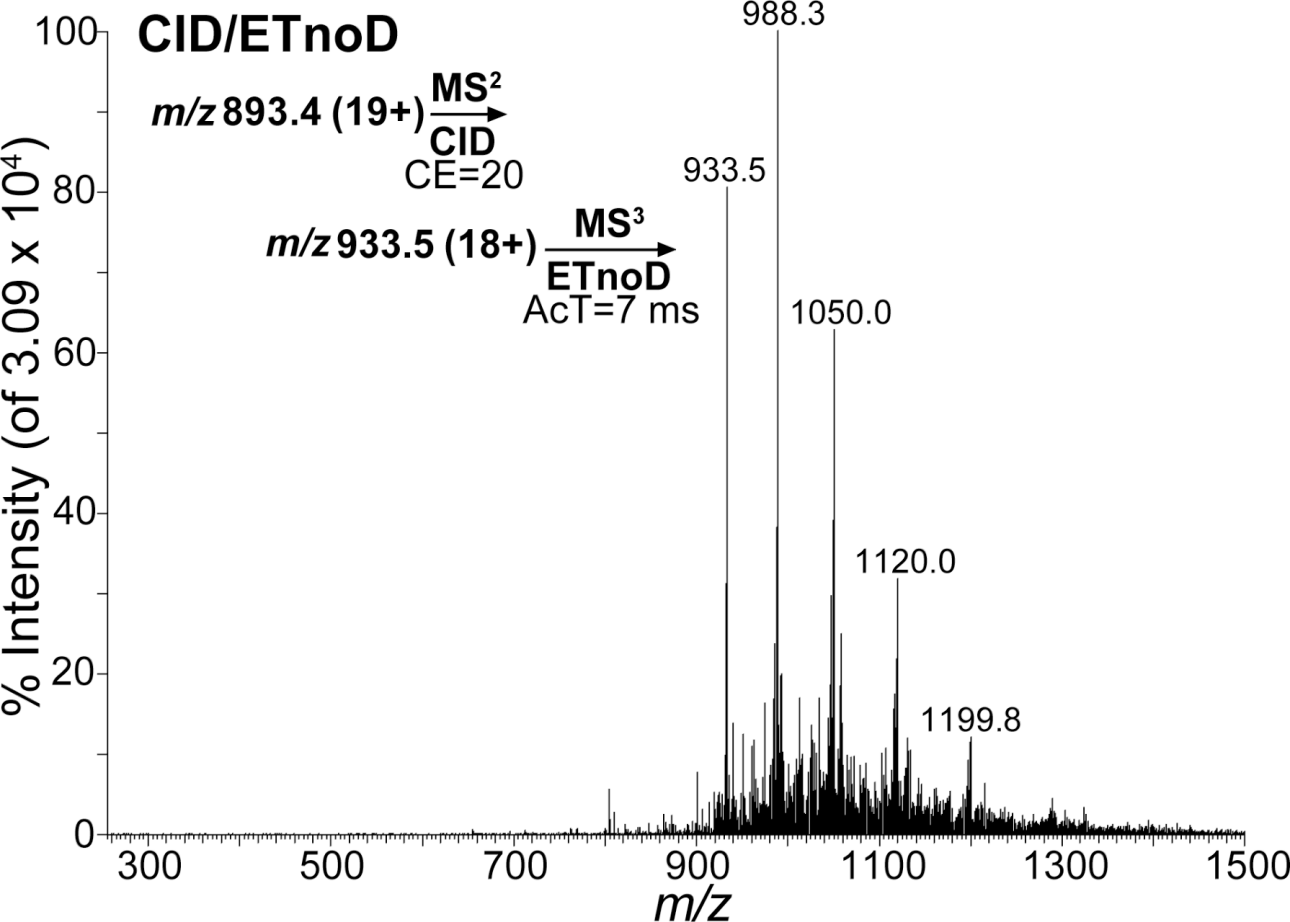
Product ion scan of intact myoglobin at collision energy (CE) = 20 and after activation time (AcT) = 7 ms (CRI observed). CID, collision-induced dissociation, ETnoD, electron transfer without dissociation; MS, mass spectrometry.

Based on the results shown in Figure 1d, the product ions (*m*/*z* 933.5), their corresponding molecular weights, and the number of charged ions could not be determined. To understand the product ions generated from intact Myo after fragmentation using the MS^3^ spectrum, the ETnoD technique was applied to generate additional product ions. After applying the ETnoD method to the precursor ions in Figure 1d, the results shown in Figure 2 were obtained, containing 4 major peaks (*m*/*z* 933.5, 988.3, 1050.0 and 1120.0). Based on Figure 2, the product ion (*m*/*z* 933.5) was identified as the precursor ion, and other peaks were identified as various CRIs (*m*/*z* 988.3, 1050.0, 1120.0, etc.). To evaluate the CRIs from Myo protein, we used two nearby peaks and used the formula 933.5x–x = 988.3 (x–1) – (x–1), setting x as the charge number of Myo ions (*m*/*z* 933.5), resulting in the outcome of x = 18 (*m*/*z* 933.5 [18+]) The shift, from 19+ to 18+, combined with the small molecular weight difference relative to native intact Myo, indicates that one charged Myo ion was lost. This finding suggests that the analysis of CRIs can provide information regarding the molecular weights of targeted fragments, representing an exciting approach that may improve our understanding of the sequences of targeted fragments. To understand the fragments observed in Figure 2, the sequences of intact Myo were added to PeakView software to calculate every amino acid and its corresponding molecular weight (Table S1). Intact Myo contains 153 amino acids (not including its initiating methionine) with a molecular weight of approximately 17 kDa. The molecular weights of the amino acids in the N- and C-terminals are also shown in Table S1. By substituting the corresponding molecular weight of intact Myo (from Figure 2), we were able to evaluate and predict the fragments, as shown in Table S2. This analysis utilized two pivotal concepts: the monoisotopic mass of intact Myo and the average mass. The molecular weight of intact Myo is rather large, with a large difference between the calculated molecular weight (monoisotopic mass) and the observed molecular weight (average mass). The difference between the monoisotopic and average mass values was added to compensate for the molecular weights of each amino acid observed, revealing the loss of two amino acids, glycine (Gly, G) and leucine (Leu, L), from the N-terminus of intact Myo as the only possible outcome to describe the intact Myo fragment.

### CID/CID/ETnoD Applied to Intact Myo

**Figure 3:**
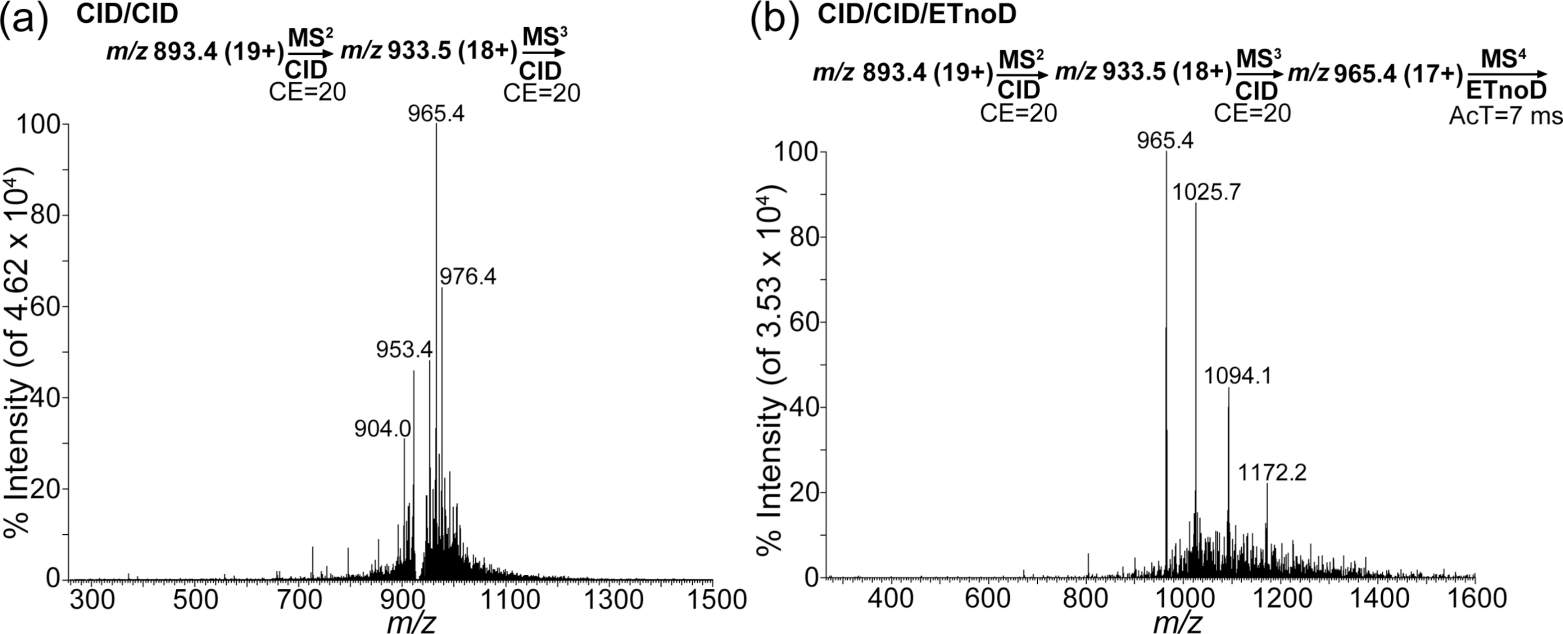
(a) Product ion scan of intact myoglobin at collision energy (CE) = 20 and after CE = 20 (one major peak observed). (b) Product ion scan of intact myoglobin at CE = 20 and after CE = 20 with activation time (AcT) = 7 ms (CRI observed). CID, collision-induced dissociation, ETnoD, electron transfer without dissociation; MS, mass spectrometry.

To confirm the predicted fragment, we applied the CID technique to the highest peak identified in Figure 1d. Figure 3a shows the treatment of the precursor ion from Figure 1d using the CID technique. The MS^3^ spectrum from Figure 3a indicates that intact Myo is fragmented twice. Although the first fragment (MS^2^ spectrum) was successfully identified in the prior analysis, the second fragment was unknown. The resulting spectrum was simple and similar to the spectrum observed in Figure 1c, but the CRIs in the major peak and their molecular weights were unknown. To evaluate the major product ions (*m*/*z* 965.4), the ETnoD method was applied as soon as the major peak in Figure 3a was observed. The results are shown in Figure 3b, which reveals the identification of the major product ion (*m*/*z* 965.4) and the various CRIs (*m*/*z* 1025.7, 1094.1, and 1172.2). To evaluate the product ions (*m*/*z* 965.4), we used the formula 965.4x–x= 1025.7 (x–1) – (x–1), in which x was the charge number of product ions (*m*/*z* 965.4), which was solved as x = 17. Identifying the number of CRIs allowed us to determine the molecular weights of the product ions in the fragmented intact Myo species (Table S3). Four amino acids from the N-terminus (serine [Ser, S], aspartic acid [Asp, D], glycine [Gly, G], and glutamic acid [Glu, E]) were cleavaged in the second fragmentation. Evidence suggests that the fragmented intact Myo in Figure 3b is part of the larger fragmented intact Myo in Figure 2. The SETRM results for the intact Myo are shown in Table 1. By analyzing the fragmented intact protein sequences and comparing them with unfragmented protein sequences, the identities of the fragments can be determined.

**Table 1:** Fragmentation of Five Intact Proteins Treated with CID^n^/ETnoD

	MS^*n*^	*m*/*z*	CE/AcT	Intensity (CID/ETnoD)	No. of AA^a^	AA changes^b^
Myo	MS^1^	893.4 ^[19+]^	N/A	5708936	153	N/A
	MS^2^	933.5 ^[18+]^	20/7	652343/24909	151	2
	MS^3^	965.4 ^[17+]^	20/7	46177/3530	147	6
Insl	MS^1^	1162.8 ^[5+]^	N/A	12103451	51	N/A
	MS^2^	1136.2 ^[5+]^	20/30	282723/52916	50	1
	MS^3^	1358.0 ^[4+]^	20/15	6324/800	47	4
Hemo	MS^1^	841.8 ^[18+]^	N/A	1962646	141^c^	N/A
	MS^2^	878.5 ^[17+]^	35/35	63433/37358	139	2
	MS^3^	967.6 ^[8+]^	35/100	2386/1088	73	68
A-lac	MS^1^	1127.7 ^[13+]^	N/A	1281838	123	N/A
	MS^2^	1238.0 ^[8+]^	35/10	159442/7423	84	39
	MS^3^	1382.2 ^[7+]^	35/10	18226/2119	82	41
Lyso	MS^1^	1137.4 ^[13+]^	N/A	1573136	129	N/A
	MS^2^	1012.2 ^[7+]^	35/10	29315/3077	63	66
	MS^3^	996.1 ^[7+]^	35/10	903/145	62	67

a Precurs or ions of all intact proteins, not including its initiating methionine.

b Total amino acid disintegration.

c Only the number of α chains in intact Hemo

### Intact Insl and Hemo

The analysis of intact Insl and Hemo was performed as described for intact Myo. Intact Insl has the smallest molecular weight among the protein standards examined in this study. The full scan of intact Insl is shown in Figure S1a. After fragmentation, the fragments released from intact Insl were identified as the b_18_ ion of the alpha-chain (second CID application) and the y_29_ ion of the beta-chain (first CID application, Figure S1b–e). We did not select the highest intensity peaks in Figure S1d (*m*/*z* 1116.1) but instead selected the peak at *m*/*z* 1358.2 because none of the sequences matched the prior peak identified in intact Insl (Table S4). Intact Insl contains three disulfide bonds, which may have affected the sequence calculations. The peak at *m*/*z* 1116.1 appears to represent a species with incompletely broken disulfide bonds, complicating the calculations. However, by selecting the high-intensity product ions at *m*/*z* 1358.2, we were able to evaluate the sequences (Figure S1e).

**Figure 4:**
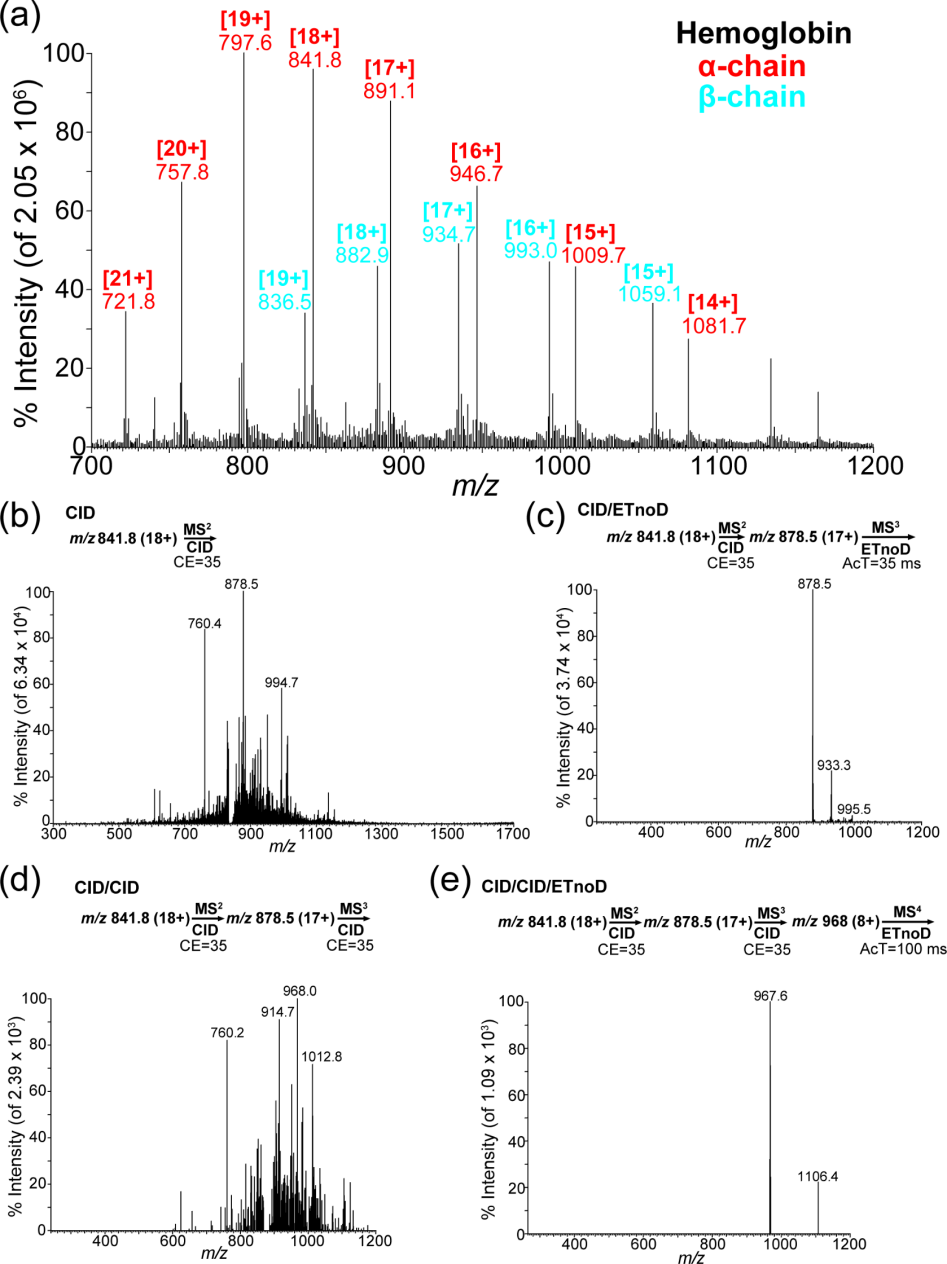
(a) Full scan of intact hemoglobin (α- and β-chains) on *m*/*z* 700 to *m*/*z* 1200. (b) Product ion scan at CE = 35. (c) Product ion scan at CE = 35 and after AcT = 35 ms. (d) Product ion scan at CE = 35 and after CE = 35. (e) Product ion scan at CE = 35 and after CE = 35 and finally AcT = 100 ms.

After acid treatment, the full scan of intact Hemo revealed the alpha- and beta-chains of intact Hemo. As shown in Figure 4a, two chains exist in the spectrum, represented by the two overlapping, mountain-like peaks. Hemo is composed of equal numbers of alpha- and beta-chains, but the alpha-chain has a higher intensity than the beta-chain, similar to previously reported results.^31^ This phenomenon makes the alpha-chain more suitable for analysis than the beta-chain. The results for intact Hemo are shown in Figure 4b-e. Figure 4c shows the loss of two amino acids, valine (Val, V) and leucine (Leu, L), from the N-terminus of intact Hemo, using the calculation in Table S5. However, after undergoing two applications of CID fragmentation, as shown in Figure 4d, a large fragment is lost (Figure 4e). As indicated in Table S5, nearly 66 amino acids were lost from the C-terminus of intact Hemo, leaving a 73-amino acid protein.

### Intact A-lac and Lyso

**Figure 5:**
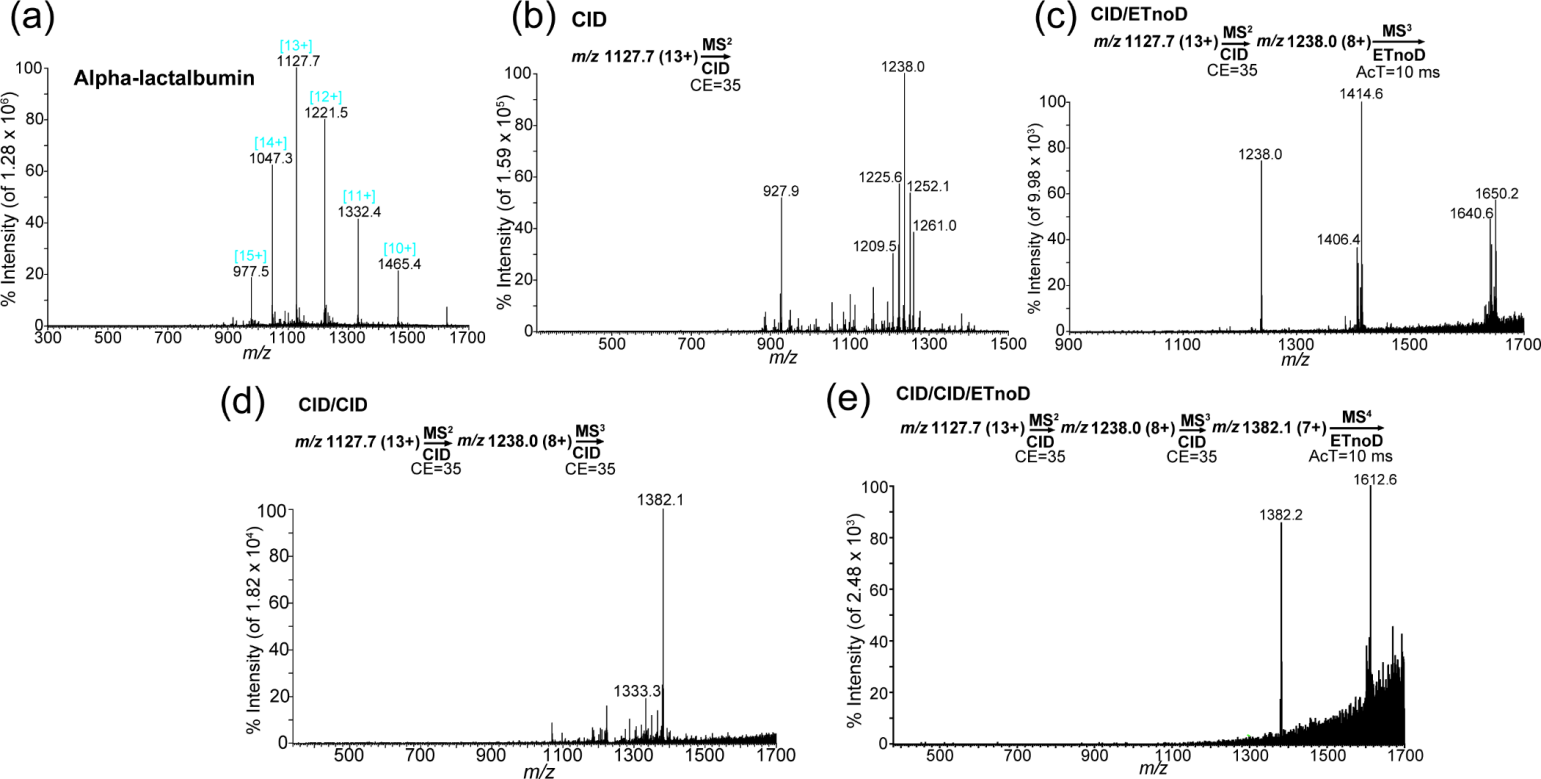
(a) Charge state distribution for intact α-lactalbumin generated by electrospray ionization and collected by full-scan mode. The highest intensity charge state [13+] was chosen as the precursor ion. (b) Product ion scan of intact α-lactalbumin at collision energy (CE) = 35 (one major stable peak observed). (c) Product ion scan of intact α-lactalbumin at CE = 35 with activation time (AcT) = 10 ms (CRI observed). (d) Product ion scan of intact α-lactalbumin at CE = 35 and after CE = 35 (one major peak observed). (e) Product ion scan of intact α-lactalbumin at CE = 35 and after CE = 35 with AcT = 10 ms (CRI observed). CID, collision-induced dissociation, ETnoD, electron transfer without dissociation; MS, mass spectrometry.

Both intact A-lac and intact Lyso had different outcomes compared with intact Myo. Both intact proteins contain four disulfide bonds, which complicated the analyses. Neither intact protein sequence was possible to predict using software (Data not shown). We used DTT to break the disulfide bonds, followed by protein stabilization using IAM. Each disulfide bond is associated with a 116.1 Da shift (two IAM molecules are added for each disulfide bond), causing each amino acid to have a 58 Da shift. These shifts cause the molecular weights of intact A-lac and Lyso to change, as shown in Table S6 and Table S7, respectively. Each intact protein reveals a nearly 464.4 Da shift due to the presence of four disulfide bonds in each protein. After the stabilization procedure, these intact proteins can be analyzed using the same methods used for the other standard proteins. The full scan results of A-lac after breaking the disulfide bonds and stabilizing the protein are shown in Figure 5a. A-lac has an increased positive charge capacity, suggesting that the presence of disulfide bonds may prevent intact proteins from becoming positively charged. The fragmentation results are shown in Figure 5b–e. In Figure 5b, the fragmentation of intact A-lac showed that nearly 39 amino acids were lost from the C-terminus, which was verified in Figure 5c and Table S8. Two additional amino acids are eliminated during later fragmentation processes, as shown in Figure 5d and verified using the ETnoD method in Figure 5e and Table S8. Figure S2a shows the full scan of the intact Lyso protein after DTT and IAM processing. The corresponding fragmentation results are shown in Figure S2b–e, and the verification is shown in Table S9.

### Five Intact Proteins Can Be Identified Using the CID^n^/ETnoD Technique

**Figure 6:**
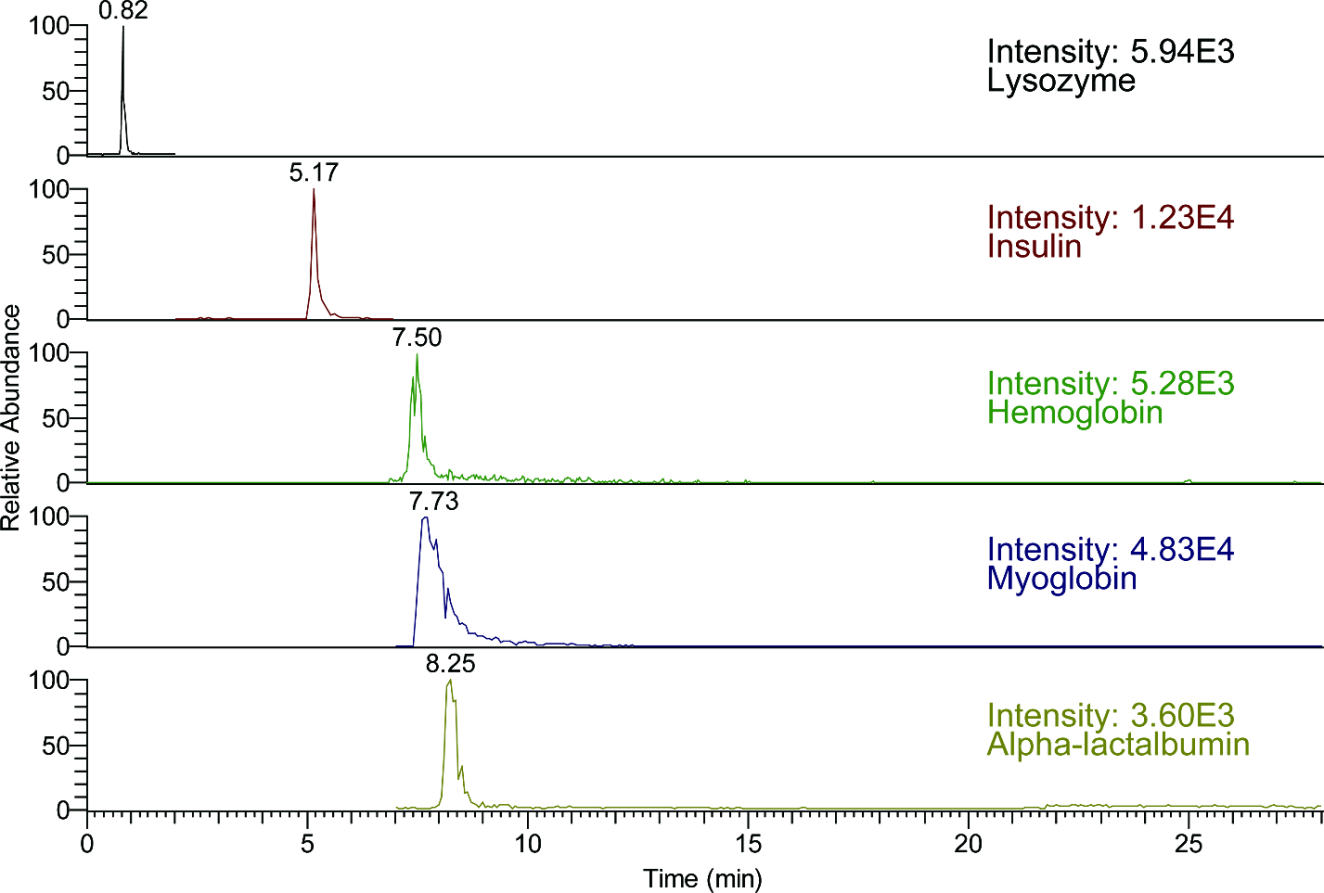
Base peak chromatography of the mixture of 5 intact proteins standards. Scheme 1 shows a simplified representation of the approaches that we used in this study. Predicting the sequences from the MS^3^ spectra may require the validation of predicted sequences provided from the MS^4^ spectra. By identifying the species in the spectra, we can identify the sequences in the remaining intact proteins. We believe that most intact proteins can be evaluated by these methods to understand the fragments generated during mass spectrometry analyses.

We successfully applied the CID^n^/ETnoD method to identify all of our evaluated intact proteins (Table 1). To our knowledge, this is the first study to use low-resolution mass spectrometry to analyze intact proteins. Only a few amino acids were fragmented in each step, allowing the missing sequences to be predicted by mass spectrometry using specific software. However, some intact proteins may require additional pretreatment steps, whereas others require the selection of different peaks to explain the resulting fragments. Disulfide bonds in intact proteins are known to complicate their analysis, and despite the use of small proteins in this study, Insl required the consideration of peak selection (Figure S 1d). However, applying slight modifications to intact proteins, such as DTT and IAM stabilization, may help us determine protein sequences. Lastly, a mixture containing five intact proteins was used to confirm the transition selected through the CID^n^/ETnoD. With the 28 min gradient, each transition presents a single peak, suggesting the specificity of each transition to the corresponding protein (Figure 6).

## Conclusion

In this work, we used CID^n^/ETnoD to predict the charges of analytes and applied it to various protein standards, obtaining satisfying results for all assessed samples. Although we only determined partial sequences for each evaluated intact protein, this study verifies that this method can be used to identify specific intact proteins in samples. We believe that this fast and direct qualification method will be beneficial for many fields and applications. Although our developed method may not be as precise as the common methods used to identify targeted peptides obtained from digested proteins, it still provides a direction for protein qualification. This quick and direct method bypasses the complications of protein digestion and the complexity of sample preparation, which should prove useful and valuable for many fields and applications. We hope that this direct and simple method can be applied across various fields to simplify sample preparation. Further work, however, remains necessary to develop methods for quantitating the corresponding intact proteins to broaden the applications of this method.

## Supporting Information Available

Mass spectra of intact Insl and Lyso in CIDn/ETnoD (PDF)
Sequence information on intact Myo (XLSX)
Fragment sequence predicting of intact Myo in CID/ETnoD (XLSX)
Fragment sequence predicting of intact Myo in CID/CID/ETnoD (XLSX)
Sequence information on intact Insl (XLSX)
Sequence information on intact Hemo (XLSX)
Sequence information on intact A-lac (XLSX)
Sequence information on intact Lyso (XLSX)
Fragment sequence predicting of intact A-lac (XLSX)
(Fragment sequence predicting of intact Lyso (XLSX)
