# Supplementary material for: Direct Identification of Intact Proteins Using a Low-Resolution Mass Spectrometer with CIDn/ETnoD
Source: J Am Soc Mass Spectrom. 2024 Jun 21;35(7):1507–15. doi: 10.1021/jasms.4c00108 (PMC11228978; doi:10.1021/jasms.4c00108)
Supplement: Supplementary file 1 — js4c00108_si_001.pdf [file js4c00108_si_001.pdf]

SUPPORTING INFORMATION

Direct Identification of Intact Proteins Using a Low-Resolution Mass Spectrometer with CIDn/ETnoD

Cheng-Yu Kuo,[1] Yi-Feng Zheng,[1] Wei-Chen Wang,[1] Jie-Teng Toh,[1] Yu-Ming Hsu,[1] Han-Ju Chien,[2] Chih-Jui Chang,[3] Chien-Chen Lai[1,4-7] *

1. Institute of Molecular Biology, National Chung Hsing University, Taichung 402, Taiwan.
2. Department of Biochemical Science and Technology, National Chiayi University, Chiayi, 600, Taiwan.
3. Department of Molecular Biology and Human Genetics, Tzu Chi University.
4. Advanced Plant and Food Crop Biotechnology Center, National Chung Hsing University, Taichung, 402, Taiwan.
5. Graduate Institute of Chinese Medical Science, China Medical University, Taichung 406, Taiwan.
6. Doctoral Program in Translational Medicine, National Chung Hsing University, Taichung, 402, Taiwan.
7. Rong Hsing Translational Medicine Research Center, National Chung Hsing University, Taichung, 402, Taiwan.

*Correspondence: Chien-Chen Lai, Institute of Molecular Biology, National Chung Hsing University, No. 250, Kuo-Kuang Road, Taichung, 40227 Taiwan.
Tel: +8864-22840485 ext. 235 Fax: +8864-22858163
Electronic address: lailai@dragon.nchu.edu.tw

Figure S1. ………………………………………………………………………………S-4

(a) Intact Insulin charge state distribution generated by electrospray ionization and collected by full scan mode.

(b) Product ion scan at CE=20.

(c) Product ion scan at CE=20 and after AcT=30 ms.

(d) Product ion scan at CE=20 and after CE=20.

(e) Product ion scan at CE=20 and after CE=20 and finally AcT=15 ms.

Figure S2. ……………………………………………………………………..…S-5

(a) Intact Lysozyme charge state distribution generated by electrospray ionization and collected by full scan mode.

(b) Product ion scan at CE=35.

(c) Product ion scan at CE=35 and after AcT=10 ms.

(d) Product ion scan at CE=35 and after CE=35.

(e) Product ion scan at CE=35 and after CE=35 and finally AcT=10 ms.

Table S1. Monoisotope b and y ions of Myoglobin

Table S2.

(a) Monoisotope b and y ions of Myoglobin

(b) Predict b and y ions from Myoglobin at CID/ETnoD

Table S3.

(a) Monoisotope b and y ions of Myoglobin

(b) Predict b and y ions from Myoglobin at CID/CID/ETnoD

Table S4.

(a) Monoisotope b and y ions of Insulin chain A

(b) Monoisotope b and y ions of Insulin chain B

(c) Predict b and y ions of Insulin at CID/ETnoD

(d) Monoisotope b and y ions of Insulin chain A

(e) Monoisotope b and y ions of Insulin chain B

(f) Predict b and y ions of Insulin at CID/CID/ETnoD

Table S5.

(a) Monoisotope b and y ions of Hemoglobin α-chain

(b) Predict b and y ions from Hemoglobin α-chain at CID/ETnoD

(c) Monoisotope b and y ions of Hemoglobin α-chain

(d) Predict b and y ions from Hemoglobin α-chain at CID/CID/ETnoD

Table S6. Monoisotope b and y ions of Alpha-lactalbumin

Table S7. Monoisotope b and y ions of Lysozyme

Table S8.

(a) Monoisotope b and y ions of Alpha-lactalbumin

(b) Predict b and y ions from Alpha-lactalbumin at CID/ETnoD

(c) Monoisotope b and y ions of Alpha-lactalbumin

(d) Predict b and y ions from Alpha-lactalbumin at CID/CID/ETnoD

Table S9.

(a) Monoisotope b and y ions of Lysozyme

(b) Predict b and y ions from Lysozyme at CID/ETnoD

(c) Monoisotope b and y ions of Lysozyme

(d) Predict b and y ions from Lysozyme at CID/CID/ETnoD

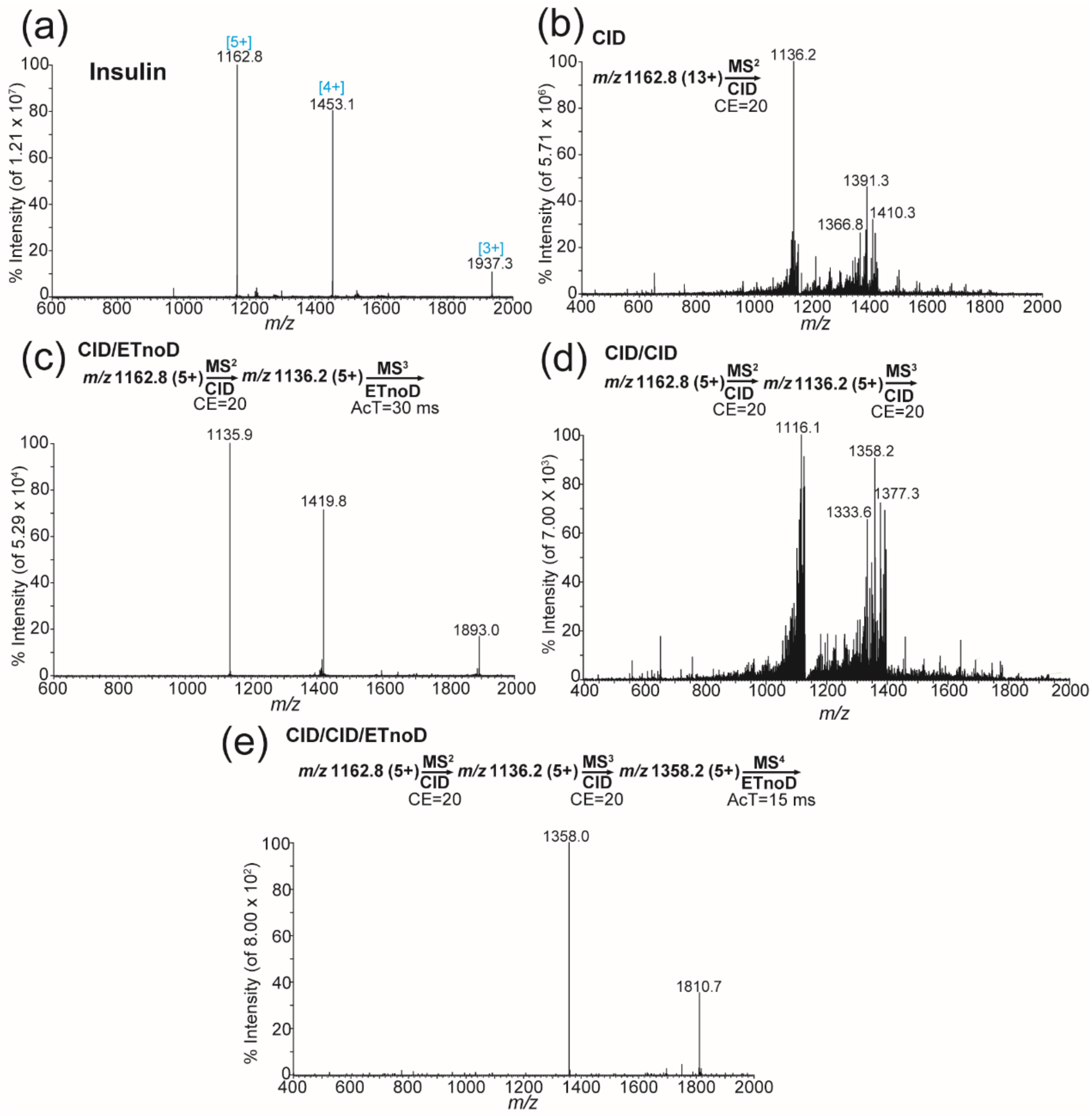

Figure S1. (a) Intact Insulin charge state distribution generated by electrospray ionization and collected by full scan mode. (b) Product ion scan at CE=20. (c) Product ion scan at CE=20 and after AcT=30 ms.(d) Product ion scan at CE=20 and after CE=20. (e) Product ion scan at CE=20 and after CE=20 and finally AcT=15 ms.

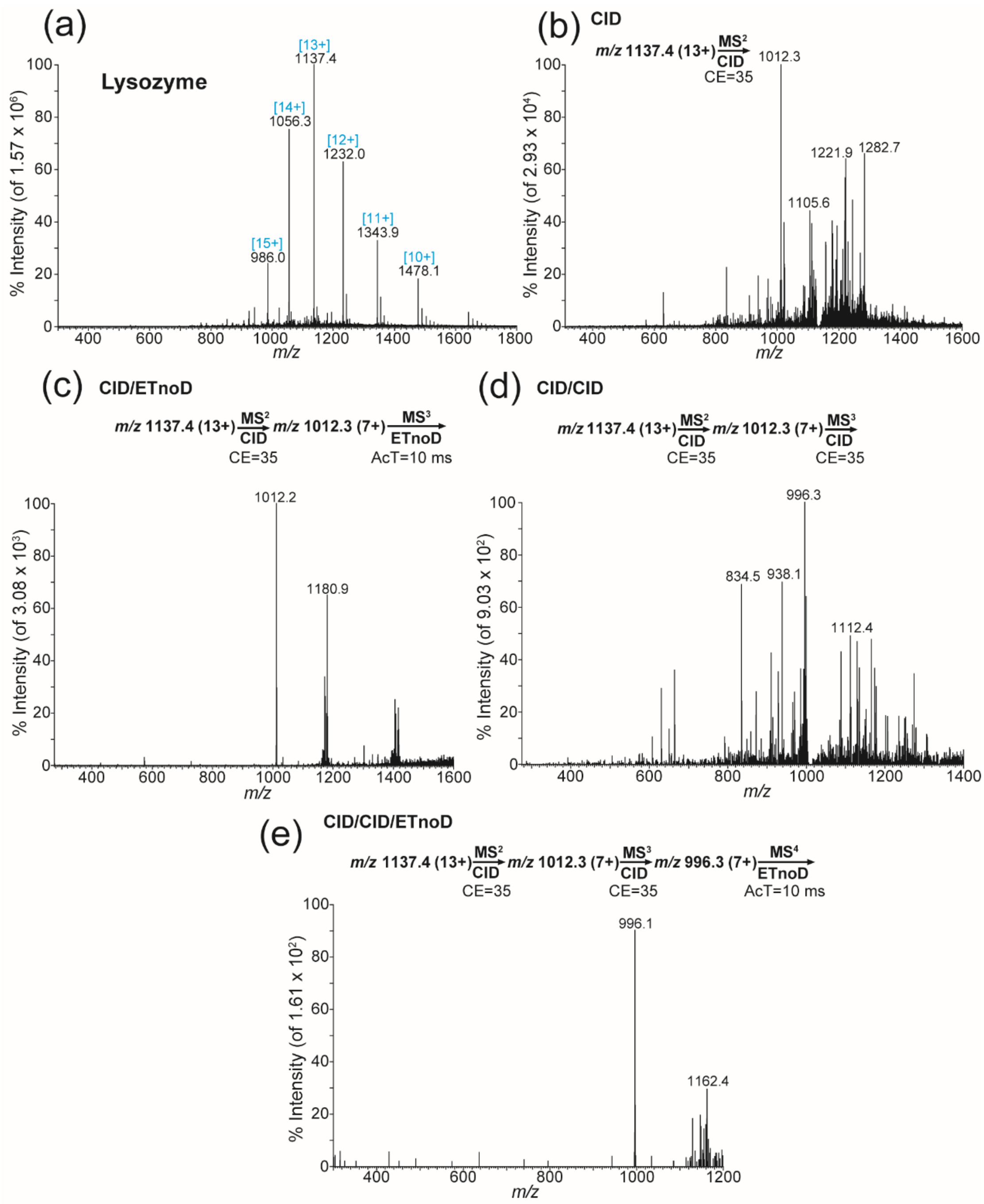

Figure S2. (a) Intact Lysozyme charge state distribution generated by electrospray ionization and collected by full scan mode. (b) Product ion scan at CE=35. (c) Product ion scan at CE=35 and after AcT=10 ms. (d) Product ion scan at CE=35 and after CE=35. (e) Product ion scan at CE=35 and after CE=35 and finally AcT=10 ms.
